# Bis(1*H*-benzimidazol-1-yl)methane monohydrate

**DOI:** 10.1107/S1600536811041572

**Published:** 2011-10-12

**Authors:** Tao Shi, Shouwen Jin, Jianlong Zhu, YingJia Liu, ChuanChuan Shi

**Affiliations:** a Faculty of Science, ZheJiang A & F University, Lin’An 311300, People’s Republic of China; b Tianmu College of ZheJiang A & F University, Lin’An 311300, People’s Republic of China

## Abstract

In the title compound, C_15_H_12_N_4_·H_2_O, the organic mol­ecule displays approximate non-crystallographic twofold symmetry: the dihedral angle between the benzimidazole ring systems is 81.37 (12)°. In the crystal, the components are linked by O—H⋯N hydrogen bonds, forming chains propagating in [101]. Aromatic π–π stacking [centroid–centroid separation = 3.595 (2) Å] helps to consolidate the structure.

## Related literature

For background to coordination polymers containing bridged imidazole systems, see: Jin & Chen (2007 ▶); Ma *et al.* (2003 ▶). For the synthesis, see: Lavandera *et al.* (1988 ▶).
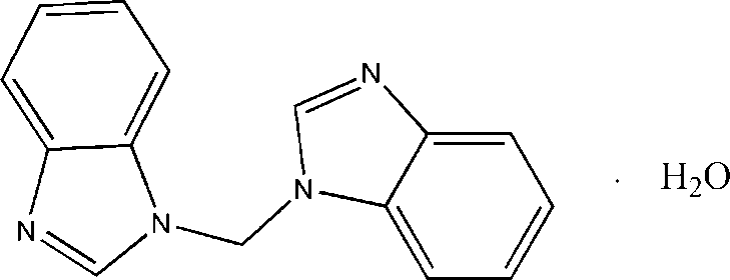


## Experimental

### 

#### Crystal data

C_15_H_12_N_4_·H_2_O
*M* *_r_* = 266.30
Triclinic, 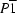

*a* = 7.3035 (6) Å
*b* = 8.9731 (8) Å
*c* = 11.1943 (10) Å
α = 103.578 (2)°
β = 103.408 (2)°
γ = 96.934 (1)°
*V* = 681.67 (10) Å^3^
*Z* = 2
Mo *K*α radiation
μ = 0.09 mm^−1^
*T* = 298 K
0.40 × 0.38 × 0.23 mm

#### Data collection

Bruker SMART CCD diffractometer
Absorption correction: multi-scan (*SADABS*; Bruker, 2002 ▶) *T*_min_ = 0.966, *T*_max_ = 0.980
3472 measured reflections
2350 independent reflections
1280 reflections with *I* > 2σ(*I*)
*R*_int_ = 0.026

#### Refinement

*R*[*F* ^2^ > 2σ(*F* ^2^)] = 0.057
*wR*(*F* ^2^) = 0.181
*S* = 1.03
2350 reflections
181 parameters
H-atom parameters constrained
Δρ_max_ = 0.20 e Å^−3^
Δρ_min_ = −0.23 e Å^−3^

### 

Data collection: *SMART* (Bruker, 2002 ▶); cell refinement: *SAINT* (Bruker, 2002 ▶); data reduction: *SAINT*; program(s) used to solve structure: *SHELXS97* (Sheldrick, 2008 ▶); program(s) used to refine structure: *SHELXL97* (Sheldrick, 2008 ▶); molecular graphics: *SHELXTL* (Sheldrick, 2008 ▶); software used to prepare material for publication: *SHELXTL*.

## Supplementary Material

Crystal structure: contains datablock(s) global, I. DOI: 10.1107/S1600536811041572/hb6425sup1.cif

Structure factors: contains datablock(s) I. DOI: 10.1107/S1600536811041572/hb6425Isup2.hkl

Supplementary material file. DOI: 10.1107/S1600536811041572/hb6425Isup3.cml

Additional supplementary materials:  crystallographic information; 3D view; checkCIF report

## Figures and Tables

**Table 1:** Hydrogen-bond geometry (Å, °)

*D*—H⋯*A*	*D*—H	H⋯*A*	*D*⋯*A*	*D*—H⋯*A*
O1—H1*D*⋯N4^i^	0.85	2.14	2.940 (4)	157
O1—H1*C*⋯N2^ii^	0.85	2.12	2.923 (3)	157

Symmetry codes: (i) 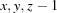; (ii) 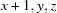.

**Fig. 1.**
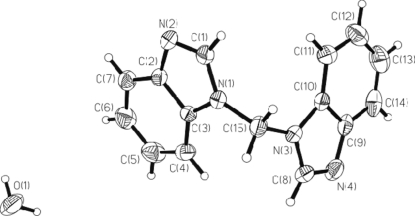
The structure of the title compound, showing displacement ellipsoids drawn at the 30% probability level.

**Fig. 2.**
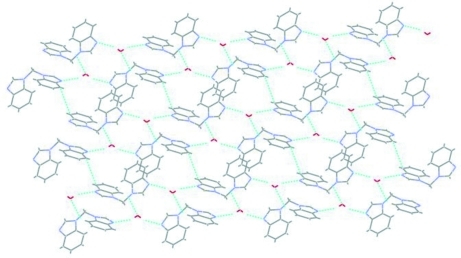
Two-dimensional corrugated sheet structure formed through π-π interactions.

## supplementary crystallographic information

### Comment

Bridged imidazole derivatives are a good choice of a N-donor ligand, and the flexible nature of the spacers allows the ligands to bend and rotate when coordinating to metal centers so as to conform to the coordination geometries of the metal ions. Significant progress has been achieved by us (Jin *et al.*, 2007) and others (Ma *et al.*, 2003) in this area.

As an extension of our research in bridged imidazole derivatives, here in this paper, we report the structure of the title compound, (I).

The r.m.s. deviations of the two benzimidazole rings are 0.003 Å and 0.007Å. They make dihedral angle of 81.37 (12)° with each other, indicating the almost perpendicular arrangement of both rings.

In the crystal, the water molecule and the bis(*N*-benzimidazolyl)methane molecule are connected together by the O—H···N hydrogen bonds to form a chain.

### Experimental

The starting material bis(*N*-benzimidazolyl)methane was prepared according to the published procedure (Lavandera *et al.*, 1988). Crystals of bis(*N*-benzimidazolyl)methane monohydrate were formed during an experiment to recrystallize the title compound. A solid of bis(*N*-benzimidazolyl)methane (24.8 mg, 0.10 mmol) in 4 ml of dmf and 1 ml of water was stirred for about 1 h at room temperature to dissolve it, then the solution was filtered into a test tube. The solution was left standing at room temperature for three weaks, colorless block crystals were isolated after slow evaporation of the solution in air at ambient temperature. The crystals were collected and dried in air to give the title compound.

### Refinement

H atoms bonded to the O atoms were located in a difference Fourier map, the O—H distance was kept 0.85 Å and refined isotropically. Other H atoms were positioned geometrically with C—H = 0.93–0.97 Å, and constrained to ride on their parent atoms with *U*_iso_(H) = 1.2 *U*_eq_(C).

### Crystal data

**Table:**

C_15_H_12_N_4_·H_2_O	*Z* = 2
*M**_r_* = 266.30	*F*(000) = 280
Triclinic, *P*1	*D*_x_ = 1.297 Mg m^−^^3^
Hall symbol: -P 1	Mo *K*α radiation, λ = 0.71073 Å
*a* = 7.3035 (6) Å	Cell parameters from 733 reflections
*b* = 8.9731 (8) Å	θ = 2.4–21.4°
*c* = 11.1943 (10) Å	µ = 0.09 mm^−^^1^
α = 103.578 (2)°	*T* = 298 K
β = 103.408 (2)°	Block, colorless
γ = 96.934 (1)°	0.40 × 0.38 × 0.23 mm
*V* = 681.67 (10) Å^3^	

### Data collection

**Table:**

Bruker SMART CCD diffractometer	2350 independent reflections
Radiation source: fine-focus sealed tube	1280 reflections with *I* > 2σ(*I*)
graphite	*R*_int_ = 0.026
φ and ω scans	θ_max_ = 25.0°, θ_min_ = 2.4°
Absorption correction: multi-scan (*SADABS*; Bruker, 2002)	*h* = −7→8
*T*_min_ = 0.966, *T*_max_ = 0.980	*k* = −9→10
3472 measured reflections	*l* = −13→12

### Refinement

**Table:**

Refinement on *F*^2^	Primary atom site location: structure-invariant direct methods
Least-squares matrix: full	Secondary atom site location: difference Fourier map
*R*[*F*^2^ > 2σ(*F*^2^)] = 0.057	Hydrogen site location: inferred from neighbouring sites
*wR*(*F*^2^) = 0.181	H-atom parameters constrained
*S* = 1.03	*w* = 1/[σ^2^(*F*_o_^2^) + (0.0662*P*)^2^ + 0.2485*P*] where *P* = (*F*_o_^2^ + 2*F*_c_^2^)/3
2350 reflections	(Δ/σ)_max_ < 0.001
181 parameters	Δρ_max_ = 0.20 e Å^−^^3^
0 restraints	Δρ_min_ = −0.23 e Å^−^^3^

### Special details

**Table:**

Geometry. All e.s.d.'s (except the e.s.d. in the dihedral angle between two l.s. planes) are estimated using the full covariance matrix. The cell e.s.d.'s are taken into account individually in the estimation of e.s.d.'s in distances, angles and torsion angles; correlations between e.s.d.'s in cell parameters are only used when they are defined by crystal symmetry. An approximate (isotropic) treatment of cell e.s.d.'s is used for estimating e.s.d.'s involving l.s. planes.
Refinement. Refinement of *F*^2^ against ALL reflections. The weighted *R*-factor *wR* and goodness of fit *S* are based on *F*^2^, conventional *R*-factors *R* are based on *F*, with *F* set to zero for negative *F*^2^. The threshold expression of *F*^2^ > σ(*F*^2^) is used only for calculating *R*-factors(gt) *etc*. and is not relevant to the choice of reflections for refinement. *R*-factors based on *F*^2^ are statistically about twice as large as those based on *F*, and *R*- factors based on ALL data will be even larger.

### Fractional atomic coordinates and isotropic or equivalent isotropic displacement parameters (Å^2^)

**Table:**

	*x*	*y*	*z*	*U*_iso_*/*U*_eq_	
N1	−0.0524 (4)	0.0854 (3)	0.6722 (2)	0.0493 (7)	
N2	−0.2207 (4)	0.1766 (3)	0.5192 (2)	0.0608 (8)	
N3	0.0643 (4)	0.1058 (3)	0.8964 (2)	0.0519 (7)	
N4	0.2853 (4)	0.2315 (4)	1.0804 (3)	0.0759 (9)	
O1	0.5456 (4)	0.1029 (3)	0.2553 (2)	0.0940 (9)	
H1C	0.6008	0.1485	0.3334	0.113*	
H1D	0.4776	0.1620	0.2238	0.113*	
C1	−0.2270 (5)	0.0924 (4)	0.6003 (3)	0.0593 (9)	
H1	−0.3411	0.0421	0.6079	0.071*	
C2	−0.0259 (5)	0.2298 (4)	0.5402 (3)	0.0488 (8)	
C3	0.0822 (4)	0.1746 (3)	0.6359 (3)	0.0445 (7)	
C4	0.2790 (5)	0.2106 (4)	0.6748 (3)	0.0642 (10)	
H4	0.3493	0.1728	0.7380	0.077*	
C5	0.3680 (6)	0.3060 (5)	0.6156 (4)	0.0838 (12)	
H5	0.5008	0.3343	0.6404	0.101*	
C6	0.2613 (6)	0.3602 (5)	0.5194 (4)	0.0806 (12)	
H6	0.3252	0.4224	0.4805	0.097*	
C7	0.0655 (6)	0.3244 (4)	0.4807 (3)	0.0637 (10)	
H7	−0.0041	0.3617	0.4170	0.076*	
C8	0.2462 (5)	0.1256 (5)	0.9711 (3)	0.0686 (10)	
H8	0.3356	0.0683	0.9463	0.082*	
C9	0.1143 (5)	0.2873 (4)	1.0789 (3)	0.0583 (9)	
C10	−0.0250 (4)	0.2100 (4)	0.9655 (3)	0.0482 (8)	
C11	−0.2091 (5)	0.2400 (4)	0.9394 (3)	0.0622 (9)	
H11	−0.3005	0.1871	0.8634	0.075*	
C12	−0.2505 (6)	0.3520 (5)	1.0315 (4)	0.0808 (12)	
H12	−0.3732	0.3750	1.0175	0.097*	
C13	−0.1141 (8)	0.4315 (5)	1.1446 (4)	0.0867 (13)	
H13	−0.1473	0.5066	1.2045	0.104*	
C14	0.0688 (7)	0.4015 (5)	1.1699 (3)	0.0788 (12)	
H14	0.1599	0.4558	1.2456	0.095*	
C15	−0.0163 (5)	0.0014 (4)	0.7692 (3)	0.0576 (9)	
H15A	−0.1356	−0.0618	0.7664	0.069*	
H15B	0.0713	−0.0682	0.7505	0.069*	

### Atomic displacement parameters (Å^2^)

**Table:**

	*U*^11^	*U*^22^	*U*^33^	*U*^12^	*U*^13^	*U*^23^
N1	0.0491 (16)	0.0624 (17)	0.0391 (14)	0.0136 (13)	0.0125 (12)	0.0168 (13)
N2	0.0571 (19)	0.087 (2)	0.0422 (15)	0.0220 (15)	0.0100 (13)	0.0243 (15)
N3	0.0533 (17)	0.0689 (18)	0.0401 (14)	0.0216 (14)	0.0122 (13)	0.0225 (13)
N4	0.065 (2)	0.112 (3)	0.0501 (18)	0.0118 (18)	0.0035 (15)	0.0338 (19)
O1	0.103 (2)	0.098 (2)	0.0688 (16)	0.0330 (17)	−0.0108 (15)	0.0273 (15)
C1	0.049 (2)	0.085 (3)	0.0428 (18)	0.0131 (18)	0.0125 (16)	0.0165 (18)
C2	0.056 (2)	0.054 (2)	0.0377 (16)	0.0181 (16)	0.0131 (15)	0.0107 (15)
C3	0.0490 (19)	0.0477 (18)	0.0417 (16)	0.0188 (15)	0.0159 (14)	0.0132 (14)
C4	0.050 (2)	0.079 (3)	0.071 (2)	0.0227 (18)	0.0155 (18)	0.031 (2)
C5	0.057 (2)	0.092 (3)	0.110 (3)	0.011 (2)	0.031 (2)	0.036 (3)
C6	0.087 (3)	0.083 (3)	0.094 (3)	0.020 (2)	0.045 (3)	0.044 (2)
C7	0.081 (3)	0.065 (2)	0.057 (2)	0.025 (2)	0.026 (2)	0.0269 (18)
C8	0.056 (2)	0.103 (3)	0.062 (2)	0.033 (2)	0.0165 (19)	0.043 (2)
C9	0.066 (2)	0.069 (2)	0.0407 (18)	0.0001 (19)	0.0132 (17)	0.0239 (18)
C10	0.053 (2)	0.058 (2)	0.0396 (17)	0.0110 (16)	0.0147 (15)	0.0212 (15)
C11	0.061 (2)	0.070 (2)	0.057 (2)	0.0191 (19)	0.0156 (17)	0.0177 (18)
C12	0.083 (3)	0.079 (3)	0.096 (3)	0.029 (2)	0.046 (3)	0.025 (3)
C13	0.117 (4)	0.067 (3)	0.080 (3)	0.010 (3)	0.050 (3)	0.009 (2)
C14	0.106 (4)	0.078 (3)	0.043 (2)	−0.012 (2)	0.021 (2)	0.011 (2)
C15	0.074 (2)	0.063 (2)	0.0438 (18)	0.0218 (18)	0.0201 (16)	0.0208 (17)

### Geometric parameters (Å, °)

**Table:**

N1—C1	1.358 (4)	C5—C6	1.397 (5)
N1—C3	1.385 (4)	C5—H5	0.9300
N1—C15	1.455 (4)	C6—C7	1.368 (5)
N2—C1	1.316 (4)	C6—H6	0.9300
N2—C2	1.391 (4)	C7—H7	0.9300
N3—C8	1.362 (4)	C8—H8	0.9300
N3—C10	1.393 (4)	C9—C10	1.395 (4)
N3—C15	1.450 (4)	C9—C14	1.396 (5)
N4—C8	1.308 (4)	C10—C11	1.381 (4)
N4—C9	1.398 (4)	C11—C12	1.377 (5)
O1—H1C	0.8500	C11—H11	0.9300
O1—H1D	0.8500	C12—C13	1.387 (6)
C1—H1	0.9300	C12—H12	0.9300
C2—C7	1.394 (4)	C13—C14	1.373 (6)
C2—C3	1.403 (4)	C13—H13	0.9300
C3—C4	1.376 (4)	C14—H14	0.9300
C4—C5	1.387 (5)	C15—H15A	0.9700
C4—H4	0.9300	C15—H15B	0.9700
			
C1—N1—C3	106.7 (2)	C2—C7—H7	121.2
C1—N1—C15	126.0 (3)	N4—C8—N3	114.7 (3)
C3—N1—C15	127.3 (3)	N4—C8—H8	122.7
C1—N2—C2	103.8 (3)	N3—C8—H8	122.7
C8—N3—C10	105.8 (3)	C10—C9—C14	119.3 (3)
C8—N3—C15	126.7 (3)	C10—C9—N4	110.3 (3)
C10—N3—C15	127.4 (3)	C14—C9—N4	130.3 (3)
C8—N4—C9	103.9 (3)	C11—C10—N3	132.0 (3)
H1C—O1—H1D	108.9	C11—C10—C9	122.7 (3)
N2—C1—N1	114.2 (3)	N3—C10—C9	105.3 (3)
N2—C1—H1	122.9	C12—C11—C10	116.6 (3)
N1—C1—H1	122.9	C12—C11—H11	121.7
N2—C2—C7	129.1 (3)	C10—C11—H11	121.7
N2—C2—C3	110.7 (3)	C11—C12—C13	121.8 (4)
C7—C2—C3	120.1 (3)	C11—C12—H12	119.1
C4—C3—N1	133.1 (3)	C13—C12—H12	119.1
C4—C3—C2	122.2 (3)	C14—C13—C12	121.3 (4)
N1—C3—C2	104.7 (3)	C14—C13—H13	119.4
C3—C4—C5	117.0 (3)	C12—C13—H13	119.4
C3—C4—H4	121.5	C13—C14—C9	118.2 (4)
C5—C4—H4	121.5	C13—C14—H14	120.9
C4—C5—C6	121.1 (4)	C9—C14—H14	120.9
C4—C5—H5	119.5	N3—C15—N1	112.2 (3)
C6—C5—H5	119.5	N3—C15—H15A	109.2
C7—C6—C5	121.9 (4)	N1—C15—H15A	109.2
C7—C6—H6	119.1	N3—C15—H15B	109.2
C5—C6—H6	119.1	N1—C15—H15B	109.2
C6—C7—C2	117.6 (3)	H15A—C15—H15B	107.9
C6—C7—H7	121.2		
			
C2—N2—C1—N1	−0.4 (3)	C15—N3—C8—N4	177.6 (3)
C3—N1—C1—N2	0.6 (3)	C8—N4—C9—C10	0.2 (4)
C15—N1—C1—N2	179.9 (3)	C8—N4—C9—C14	179.9 (3)
C1—N2—C2—C7	179.9 (3)	C8—N3—C10—C11	−178.7 (3)
C1—N2—C2—C3	0.0 (3)	C15—N3—C10—C11	3.4 (5)
C1—N1—C3—C4	179.8 (3)	C8—N3—C10—C9	0.5 (3)
C15—N1—C3—C4	0.5 (5)	C15—N3—C10—C9	−177.5 (3)
C1—N1—C3—C2	−0.5 (3)	C14—C9—C10—C11	−0.9 (5)
C15—N1—C3—C2	−179.8 (3)	N4—C9—C10—C11	178.8 (3)
N2—C2—C3—C4	−180.0 (3)	C14—C9—C10—N3	179.8 (3)
C7—C2—C3—C4	0.2 (4)	N4—C9—C10—N3	−0.4 (3)
N2—C2—C3—N1	0.3 (3)	N3—C10—C11—C12	179.3 (3)
C7—C2—C3—N1	−179.6 (3)	C9—C10—C11—C12	0.2 (5)
N1—C3—C4—C5	−179.9 (3)	C10—C11—C12—C13	0.3 (5)
C2—C3—C4—C5	0.4 (5)	C11—C12—C13—C14	−0.2 (6)
C3—C4—C5—C6	−1.0 (5)	C12—C13—C14—C9	−0.5 (6)
C4—C5—C6—C7	1.1 (6)	C10—C9—C14—C13	1.0 (5)
C5—C6—C7—C2	−0.5 (6)	N4—C9—C14—C13	−178.7 (3)
N2—C2—C7—C6	−180.0 (3)	C8—N3—C15—N1	−110.3 (3)
C3—C2—C7—C6	−0.1 (5)	C10—N3—C15—N1	67.3 (4)
C9—N4—C8—N3	0.2 (4)	C1—N1—C15—N3	−112.9 (3)
C10—N3—C8—N4	−0.4 (4)	C3—N1—C15—N3	66.3 (4)

### Hydrogen-bond geometry (Å, °)

**Table:**

*D*—H···*A*	*D*—H	H···*A*	*D*···*A*	*D*—H···*A*
O1—H1D···N4^i^	0.85	2.14	2.940 (4)	157
O1—H1C···N2^ii^	0.85	2.12	2.923 (3)	157

Symmetry codes: (i) *x*, *y*, *z*−1; (ii) *x*+1, *y*, *z*.
